# Can the Discovery of High-Impact Diagnostics Be Improved by Matching the Sampling Rate of Clinical Diagnostics to the Frequency Domain of Diagnostic Information?

**DOI:** 10.3390/cancers17091387

**Published:** 2025-04-22

**Authors:** Steven W. Millward, Peng Wei, David Piwnica-Worms, Seth T. Gammon

**Affiliations:** 1 Department of Cancer Systems Imaging, The University of Texas MD Anderson Cancer Center, Houston, TX 77030, USA; smillward@mdanderson.org (S.W.M.); dpiwnica-worms@mdanderson.org (D.P.-W.); 2 Department of Biostatistics, The University of Texas MD Anderson Cancer Center, Houston, TX 77030, USA; pwei2@mdanderson.org

**Keywords:** fourier analysis, multi-resolution analysis, diagnostic imaging, drug development, product development, cell death, inflammation imaging

## Abstract

Over the past 30 years, academic and industrial research investigators have developed molecular reporters to visualize cell death in complex biological systems. In parallel, clinical researchers, chemists, biochemists, and molecular biologists have endeavored to translate these molecular tools into clinical imaging agents. Despite these efforts, there are no clinically approved imaging methodologies with which to image cell death consistently and quantitatively. One reason may reside in the intrinsic mismatch between the sampling frequency of translational molecular imaging and the biochemical kinetics that define cell death. Beyond cell death imaging, many active research programs are now attempting to create translational diagnostic pharmaceuticals to image immunological, fibrotic, amyloidotic, and metabolic pathways. Each of these pathways is defined by a unique set of biochemical rate constants, some of which are associated with key predictive pathways. Exhaustively sampling all permutations of pathways and kinetic constants would seem to be an intractable strategy for target identification and validation. Sampling theory, if applied to these pathways, could accelerate the translation of high-impact diagnostics through prioritization of pathways for either AI enhanced diagnostic imaging or AI-enhanced wearable devices. In this perspective, we identify the Nyquist sampling rate as a key criterion for evaluating the optimal application for novel diagnostics. Sampling theory states that to fully characterize a band-limited, stationary, temporal data set, the signal must be sampled at more than twice the rate of the fastest frequency in the signal or, for diagnostics, the discriminatory signal. Through the study of the medical imaging process chain, Nyquist sampling rates of 0.25 day^−1^ and, more likely, slower than 0.02 day^−1^ were determined to provide high quality information. By prioritizing low-frequency predictive processes, or “state changes,”, imaging researchers may improve the “hit rate” of research programs by appropriately matching the rate of change in diagnostic and predictive information with the limiting sampling rate of medical imaging. Critically, however, high-frequency diagnostic information (and therefore high-frequency biological processes) need not be ignored; these processes are simply better interrogated through continuous monitoring, e.g., by wearable devices combined with machine learning or artificial intelligence.

## Simple Summary

Development of pure diagnostics, particularly injectable diagnostics, requires years of effort and a high cost to both the public and private sector. There is a nearly inexhaustible number of biochemical pathways or combinations of pathways that could be targeted to yield information on the disease state. Simultaneously, there is an explosion of wearable devices providing “continuous” readouts that are being coupled with machine learning and artificial intelligence (AI). Through the study of the time constants associated with medical imaging process chains, a method for prioritizing targets for either medical imaging or continuous device-mediated readout is proposed with the long-term goal of building high-value diagnostics to improve patient outcomes.

## 1. Introduction

For more than 30 years, numerous research groups have worked to develop molecular reporters to visualize cell death (e.g., apoptosis, necrosis) in complex biological systems. In parallel, clinical researchers, chemists, biochemists, and molecular biologists have endeavored to translate these molecular tools into clinical imaging agents. Despite these efforts, there are no clinically approved imaging methodologies that can interrogate this fundamental process consistently and quantitatively. 

First, it is critical to recognize that the goal of a diagnostic is to provide novel and actionable information by reducing the uncertainty of predicting a future event. The predictive power of a diagnostic is increased as this uncertainty is decreased. Improving predictive power is related to, but distinct from, quantifying biochemical processes, compartments, or rate constants. Furthermore, improved predictive power for a given future event must enable a care decision that improves patient outcomes relative to the cost of imaging. Optimization of the patient outcomes and the economics of diagnostics is also critical, but beyond the scope of this perspective. 

These constraints underpin the challenge of developing new clinical diagnostic imaging procedures. Moreover, unlike conventional fluid diagnostics and liquid biopsies, imaging-based diagnostics yield a combination of both spatial and temporal data across a variety of scales. Importantly, all spatio-temporal data, particularly those that involve sampling complex or fractal systems (e.g., organisms or ecosystems) display scale-dependent properties [1,2]. As an example, observed changes at the micrometer scale may be poorly predictive of disease state, but changes at the centimeter or meter scale may be highly predictive despite equivalence of measurement type and precision. Similarly, predictive changes present at the microsecond scale may be entirely absent at the minute scale (and vice versa). Well-established literature [3] studies the effect of incidence rate on biomarkers, diagnostics, and clinical trial design. Only a small subset of papers discusses the importance of temporal domain sampling in medical diagnostics, and those that do revolve either around a deep review of the apoptosis literature in imaging, exercise physiology, cardiology, or rate constant quantification [4,5,6,7,8]. In materials sciences, atmospheric science, and engineering (failure mode analysis), the effects of sampling frequency on spatio-temporal data are more deeply explored [9,10,11].

The mismatch between the kinetics of molecular imaging sampling frequency and the kinetics of information-rich biochemical pathways is clearly seen in the case of programmed cell death [4]. This mismatch particularly confounds measurement of therapy-induced cell death against a substantial background of spontaneous and heterogenous (non-induced) cell death. Only integrator systems, where either the reporter slowly clears, or a rapidly clearing reporter reports on an accumulation (or integration) of cell death products and permeabilization, show promise in cell death imaging [12,13]. Thanks to the success of immunotherapy in cancer treatment, translational diagnostic pharmaceuticals that image immunological, fibrotic, amyloidotic, and metabolic pathways are under active development from pre-clinical to clinical stage [14,15,16,17]. Are there generalizable lessons from cell death imaging in oncology that could accelerate these research programs? 

## 2. Review of Rate Constants in the Biological Systems

Consider the course of immune response and wound healing—a well-studied, spatio-temporally regulated process in both humans and animals. The kinetics of the normal healthy process are log-based, phased time series that follow a pattern of perturbation, reaction, and return to homeostasis. First, resident members of the innate immune system (mast cells, basophils, eosinophils, and macrophages) react to insults with time constants set by phosphorylation signaling cascades over seconds to minutes [18]. Migratory innate immune cells, such as neutrophils, eosinophils, and monocytes, then begin to localize and activate. Interestingly, local recruitment of neutrophils appears to be tightly conserved, with peaks occurring at 24–48 h following a variety of pathological insults [19]. Concurrent secretion of cytokines and chemokines into the blood and lymphatic system recruits cellular members of the adaptive immune system. Adaptive immune cells target pathogens directly or indirectly while creating long-term memory over days to weeks. Finally, members of the innate immune system complete the wound healing process by degrading the local fibrotic field over days to months [20]. 

Disease states occur when the phase, magnitude [21], or induction of this process is dysregulated, often through changes in key biochemical networks downstream of epigenetic recoding and microRNA deregulation. For example, during fibrosis, chronic changes in both metabolism and signaling through the TNFα and TGF-β pathways lead to consistent overproduction of collagen fibers [22]. Redox electron migration in solution occurs on microsecond time scales [23], intracellular calcium transients occur on microsecond to millisecond time scales (and, interestingly, can also exhibit predictive changes on longer time scales) [24], gene expression changes occur over minutes to hours, and cell division occurs on the time scale of one to several days. Biological and biophysical feedback mechanisms connect all scales; thus, these systems are rarely stationary and display powerful autocorrelation features (although electrocardiogram activity, some circadian rhythms, and hormonal cycles are notable exceptions) [25]. A continuous mismatch between the rate constants of production of these collagen fibers, their cross-linking, and their degradation by members of the innate immune system yields fibrosis, scarring, and potentially tumorigenesis. Given the parallels between the timing challenges in imaging cell death and imaging immune activity, there may be general principles that will facilitate the development of novel imaging-based clinical diagnostics. 

## 3. Structure, Decomposition, and Study of Spatio-Temporal Data Sets 

The continuous, multiscale, and decomposable properties of space and time provide nearly inexhaustible opportunities to improve patient outcomes through permutations of biological, biochemical, or physical variables sampled over multiple spatio-temporal scales. Therefore, researchers have developed a variety of methodologies to decompose complex spatio-temporal data sets into different spatial and temporal scales. These include Fourier analysis for stationary systems (rarely clinically relevant) to multi-resolution analysis (MRA) methods such as wavelet transforms [26] for nonstationary systems. MRA transforms enable the quantification and representation of continuous variables at distinct locations and scales in both space and time. Choosing the wrong pair can lead to technically sophisticated but fundamentally unpredictive or unactionable tools with limited clinical impact. While the ability of diagnostic imaging to couple spatial and temporal data is unique, a priori prediction of the relevance of any variable/scale pairing is often poor. While a deterministic a priori selection of relevant variables and scales may not be possible, an examination of the process chain of diagnostic medical imaging, with its inherently limited sampling rates in the temporal dimension, may help focus research efforts on more readily translatable solutions. 

Information theory provides a guide for prioritizing the choice of time scale and target. Identification of the Nyquist sampling rate for medical imaging provides a key criterion for assessing new targets for diagnostic imaging. Sampling theory states that to fully characterize a band-limited, stationary, temporal data set, the signal must be sampled at greater than twice the rate of the fastest frequency in the signal. This critical frequency is the Nyquist frequency. In addition to oscillatory systems (i.e., complex exponentials), Nyquist theory can also be applied to real-valued exponentials. For example, biological and biochemical decay processes often follow exponentials, and tumor growth initially also follows an exponential. Like oscillators, real-valued exponentials must be sampled at greater than twice the decay rate of the fastest exponential of interest. 

As indicated in Section 2 above, most medically relevant biological processes consist of layers of information generated by interacting members at multiple time scales. Therefore, biological systems are often better modeled or studied by MRA than by Fourier analysis. MRA decomposes temporal (or spatial) data into a multidimensional representation of both time and frequency. An analogy is the common Western musical notation used to produce symphonies. Time is recorded on the horizontal axis, frequency on the vertical axis, and each instrument’s role encoded on a separate but related sheet of music. Thus, MRA was a natural and successful choice for compression and detection in musical and voice data applications [27,28]. Like Fourier analysis, the choice of sampling rate in discrete MRA is critical [27]. The choice of when to sample the system and how to sample the system affects both the accuracy of the reconstruction and the quality of algorithmic training. Furthermore, a clinical diagnosis must confront a high hurdle. It is not enough to characterize a biological or biophysical process. The lifetime or frequency domain of the diagnostic or discriminatory information must be well characterized by the sampling frequency. Thus, if a limiting sampling rate for imaging-based diagnostics can be identified, researchers could prioritize targets based upon a priori knowledge of either their fluctuation frequencies or the frequency domain of their predictive information. Similarly, this would facilitate de-prioritization of targets with high variance or targets with predictive power at frequencies markedly faster than the limiting rate.

## 4. Identification of Limiting Sampling Rate in the Clinical Practice of Diagnostic Imaging

**Figure 1:**
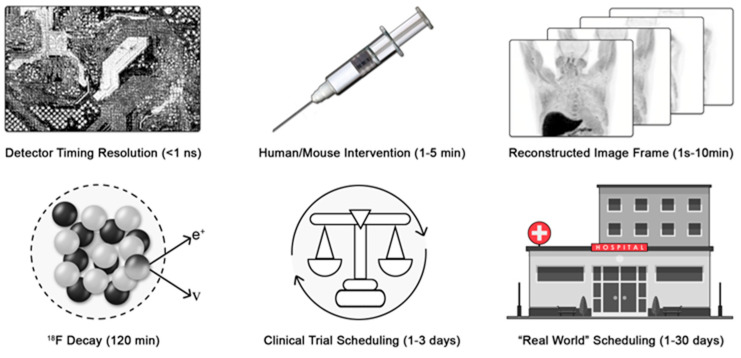
Variance in patient scheduling limits the time resolution of medical imaging. The process chain of deploying a medical diagnostic must be considered to identify the limiting rate of the process sampling frequency. e^+^ is a positron and ν is a neutrino.

Can we identify the Nyquist sampling rate for medical imaging processes? Consider positron emission tomography (PET) and X-ray computed tomography (CT) as exemplar medical imaging processes. To determine coincident events, modern detector electronics sample the 511 keV annihilation photons emitted from the patient at the picosecond time scale to generate imaging data. The tissue clearance of an injectable radiolabeled small molecule typically occurs with rate constants from minutes to hours. F-18 decays with a half-life of approximately 120 min. Thus, a practitioner would wait at least five to ten times the half-life of decay of the F-18 before re-injecting another radiotracer and re-imaging another pathway (Figure 1). 

Finally, the imaging session must be scheduled, and the patient must arrive for imaging. For motivated patients in well-controlled clinical trials, this may occur between 24 and 72 h (median expectation of 48 h). However, in broader clinical practice, the variance in scheduling is significantly greater. Scheduling requires various levels of private or public pre-authorization. Furthermore, the availability of imaging depends on the healthcare delivery system, location, needs of the patient, and constraints of the delivery system. Quantitative wait-time data are sparse, but in a global survey heavily weighted toward major US cities, wait times ranged from 1 to 6 days for PET [29] (Figure 1). More recent data from the United Kingdom found wait times of 3.3 weeks for magnetic resonance imaging and 2.5 weeks for CT [30]. Provisionally, we will estimate that reliable imaging occurs with a maximum sampling rate of 0.5 day^−1^ and more often as slow as 0.04 day^−1^. 

Therefore, for those seeking to develop new diagnostic imaging strategies, the aggregate rate of patient scheduling, arrival, tracer injection, and imaging becomes the limiting sampling rate for all medical imaging. Again, this result is fundamentally different from, and slower than, the achievable rate for studying biological and biochemical processes in a research laboratory setting. Quantitatively, the change or loss in diagnostic information must be slower than the Nyquist sampling rates of 0.25 day^−1^, and is more likely slower than 0.02 day^−1^. These rate constants quantitatively agree with the clinically recommended Response Evaluation Criteria in Solid Tumors (RECIST) timing for imaging of colorectal cancer at baseline and 2 months later, a sampling rate of ~0.017 day^−1^. Not coincidentally, the median exponential growth rate of colorectal cancer [31] is 0.005 days^−1^, a slow process that is well matched to the Nyquist rate of medical imaging, 0.02 day^−1^. 

## 5. Recommendations

### 5.1. Low-Frequency, Temporally Delocalized Diagnostic Information Harmonizes with Clinical Imaging

In a variety of biological processes, the transitions between health and disease occur over prolonged periods of time. In some cases, these processes exhibit higher-frequency fluctuations that are either nonpredictive or marginally predictive. Given the intrinsic uncertainty in the timing of imaging relative to changes in disease state, we propose that these lower-frequency processes are the ideal target for diagnostic imaging. First, as indicated above, these processes match the Nyquist sampling rate of medical imaging. Second, low-frequency predictive information is favorable through the lens of error analysis. For diagnostic information that changes slowly over time, large uncertainty in the timing of imaging will yield only small improvements in the predicted outcome. 

Does leveraging low frequency diagnostic information harmonize with other heavily regulated spaces in medicine? Comparing the study of chemical reactions to the characterization of a medicinal compound is an analogy that may resonate with diagnostic researchers. Many chemical transformations occur on the nanosecond or picosecond time scale; observing the extremely short-lived intermediates of these reactions requires highly sophisticated techniques [32]. These techniques provide invaluable information about ultra-fast electronic processes. but require ultra-high-frequency sampling regimes. Nuclear magnetic resonance, despite its power to resolve atomic-level changes in molecular structure, cannot be used to interrogate chemical changes on time scales faster than its comparatively slow sampling rate (seconds to milliseconds). Thus, while high-frequency sampling in controlled environments enables researchers to study chemical transition states, the transition states of compounds are rarely if ever used in applied chemical manufacturing controls (CMC) for medicinal compounds. How are these limitations relevant? CMC release criteria are diagnostic criteria for known or expected modes of future toxicity or loss of potency. A key CMC release criterion for medical compounds is the state change or degradation of a compound below a prespecified limit. Indeed, state change is so important that it is often measured with multiple, partially redundant methods. Thus, we propose that many dynamic biological processes, “cell-state transitions”, are analogous to chemical transition states. High-frequency sampling in controlled environments enables their study, but rarely yields robust, medically actionable targets for diagnostic imaging. 

It is instructive to consider the relationship between the rates of underlying process changes and the diagnostic information yielded by successful medical diagnostics. In addition to colorectal cancer imaging, discussed above, other examples include calcifications detected by mammography, broken bones, heart size, liver fat, and PET response criteria in solid tumors (PERCIST). The changes in the underlying biology and diagnostic information occur slowly and build up over time in the case of liver fat, heart size, and breast calcifications. Mammography is a phenomenally successful diagnostic tool that images both the rate of change in the density of breast tissues and the activation of the innate immune system through the additive local deposition of calcium after inflammatory activation. Functional PET imaging also detects low-frequency changes in biochemical pathways. PERCIST raises an interesting subclass in which faster local sampling may better define a slowly changing process. For example, the change in the rate of transport and phosphorylation of glucose (or analogs like FDG) aids in the diagnosis and upstaging of cancer. The change in the rate of transport varies slowly over time, but the rate constants of transport and phosphorylation can occur much more rapidly. Therefore, rapid (on the order of seconds) kinetic sampling and modeling are being studied to improve the predictive value through more precise measurements of the rate constants of transport and phosphorylation [33,34]. Similarly, in an emerging technique, slow changes in the rate of transport and metabolism of a bolus of ^13^C-pyruvate can be effectively measured by the rapid analysis of hyperpolarized magnetic resonance spectroscopy [35,36]. Interestingly, while the above diagnostic imaging methods all function at the millimeter to centimeter spatial scale, diagnostically important slow temporal changes are not restricted to macro-structures or low frequencies in the spatial domain. For instance, at the micrometer spatial scale, optical coherence tomography leverages the slow loss of retinal ganglion cells and their associated axons to help diagnose and manage the treatment of glaucoma [37].

Finally, the conversion of a rapid process into a dysregulated, slow, chronic process might also be powerfully predictive. Examples include the conversion of the cellular innate immune system from its normal, rapid, phase-locked activation and subsequent inactivation into chronic, temporally delocalized activation. The resulting chronic inflammation induces, maintains, and often exacerbates diseases such as atherosclerosis and arthritis. Because the intensities of these processes are often low relative to those of high-frequency, acute changes, researchers must develop imaging processes with large signal-to-noise ratios and effect sizes. 

Within this low-frequency temporal regime, a variety of techniques can be leveraged to maximize, automate, and discover information across various spatial frequencies contained within the data set [38,39]. While a full review is outside of the scope of this article, importantly, recent provocative large clinical trial data of more than 100,000 women demonstrate that AI can dramatically scale and deliver the value of breast cancer mammography with minimal increase in false positive effects, which are primarily captured in the aging population [40].

### 5.2. High-Frequency, Temporally Localized Diagnostic Information Harmonizes with Personalized Monitoring and Wearable Devices Coupled with Machine Learning and Artificial Intelligence

Through studying biological mechanisms or attempting to solve clinical problems, researchers may identify temporally localized high-frequency processes that are highly predictive of patient outcomes. If the rate constants of those predictive processes are faster than 0.25 day^−1^, the intrinsic slow sampling rates of medical imaging will yield substantial uncertainties and poor predictive value, as modeled for cell death. This does not imply, however, that these fast diagnostic processes are completely unapproachable. Their readout will need to occur outside of high-end, equipment-intensive medical imaging environments. Wearable devices such as those now approved in the United States and Europe for monitoring and delivery of insulin are a powerful recent example. These devices sample 4 times per day, 24 h a day, 7 days a week, ref. [41] approximately 100-fold faster than the longest sampling rate of medical imaging. Similarly, wearable heart monitors are now available for at-home patient use. From these two examples, one can also observe an interesting trade-off. For these biochemical pathways, spatial information or frequency is traded for improved temporal resolution. 

One can also imagine simple optical reflectance, fluorescence, or absorbance devices located in the patient’s home, on a smartphone, or as a wearable device. These personalized imaging devices could noninvasively interrogate high-frequency (>1 day^−1^) predictive biological processes. Furthermore, scalable personalized software and hardware can integrate high-frequency data sets into digital medical records, and machine or deep learning algorithms could leverage both high-frequency data from wearables and low-frequency data from medical imaging through integrated MRA [42]. In the United States, the most straightforward path to approval is through either a type 1 or type 2 diagnostic approval process, where appropriate demonstration of accuracy and better improvement of outcomes must be demonstrated. The cost-effectiveness and viability of these processes are yet to be determined. 

Monitoring high-frequency information can also deliver unique value to patients and the healthcare system through high-frequency reminders for intervention. In this use case, the high-frequency diagnostic information is coupled through rapid, <1 day at home or <1 h inpatient [43], delivery of the diagnostic conclusion to a provider to intervene. This has recently been tested in the critical care ward [44], and is under study to prevent suicide through sleep monitoring of psychiatric inpatients [45]. 

For at-home imaging and wearable devices, significant management of the security of patient data, both at the point of collection and transmission, must be carefully managed. For web-based applications, publicly accessible and continuously updated rankings of lists are available, such as the OWASP Top 10 [46] and NVD (National Vulnerability Database) [47]. While there are many potential solutions, the current practice for a wearable heart monitor includes leveraging the already encrypted cellular tower network with a dedicated encrypted cellular phone containing limited applications. This phone also prevents users from adding additional untested applications that could invite vulnerabilities. Several recent reviews provide additional insightful vulnerabilities and potential solutions [48,49]. 

Critically, as continued economic pressure is applied to imaging in healthcare systems [50] identifying high-value, predictive interaction terms between previously high-cost, low-predictive value, low-frequency imaging and previously low-predictive value, low-cost, high-frequency wearable data could have a dramatic and positive impact on clinical care. Multi-resolution decomposition prior to training, followed by training at specific scales and followed by MRA reconstruction, has already proven helpful in a variety of AI systems and should indeed apply to medical data records. Training of these data sets will not necessarily be trivial. The large volumes of data required for most AI systems will require ethical exploration and training in large electronic medical records or through opt-in personal devices. This will likely require either public–private partnerships between companies and large healthcare delivery systems or national healthcare delivery systems. Certain types of wearable data might be able to be acquired through opt-in monitoring on devices such as cellular phones and harmonizing that with EMR, again on an opt-in basis. Finally, the a priori selection of complementary scales from different inputs might minimize training, update, and storage times while maximizing the predictive power of AI or deep learning algorithms. For example, low-frequency temporal data from medical imaging could be selected or weighted more heavily during training, while conversely, high-frequency temporal data could be more heavily weighted from the wearable device. Furthermore, many “black box” or even “grey box” algorithms contain pre-filtering algorithms to prevent “noise”, usually machine noise, from overly influencing the resulting algorithms [51]. The accessible high-frequency information necessarily has a lower frequency than these built-in filters. Alternatively, custom algorithms could be built to capture very high-frequency data under the assumption of high signal-to-noise ratios within the high-frequency band [52]. 

Additionally, there is a rare exception: pathways that are still suited to medical imaging when predictive power lies in the high-frequency domain. That subset consists of highly phase-locked, short-rate-constant diagnostic processes. If treatment potently induces novel diagnostic information on the scale of about 12 h or more, a variety of imaging test and retest scenarios, including ^18^F-based PET, may be enabled. Thus, most phase-locked systems will remain more amenable to monitoring through wearable devices unless spatial information is truly critical.

### 5.3. Leveraging the Existing Literature or Emerging Data Without Formal Fourier or Multi-Resolution Analyis Is an Emerging Opportunity for AI

For well-studied biochemical processes, researchers may survey the literature to prioritize novel targets without measuring their predictive value. For example, a manual retrospective survey method was used to quantify the timing variability of the apoptotic machinery. As large language models and AI-assisted image interpretation become widely available, the process of data extraction from manuscripts and conversion of these extracted data into temporally resolved aggregate data sets will become easier. For less well-studied processes, researchers and large language models might also identify key “words of warning” in the literature to de-prioritize certain targets and pathways. The phrase “precise control of timing” or the word “sporadic” imply that the frequency domain of the predictive information is high or susceptible to temporal error relative to the frequency domain of sampling available to the researchers in the published study. Conversely, the phrases “robust to timing”, “robust to route of injection”, or “state change” (indicating a long-time constant process) imply potentially actionable, slow, but predictive targets that could be prioritized for translation. Finally, the identification of rapid phase-locked systems is more difficult and fraught with overpromises due to potential species shift in rate constants of response between models or model organisms and the clinic. Nonetheless, the phrase “always occurs within a week to several days” may indicate a possible phase-locked target, particularly when this statement is conserved between pre-clinical models and clinical practice.

## 6. Conclusions

**Figure 2:**
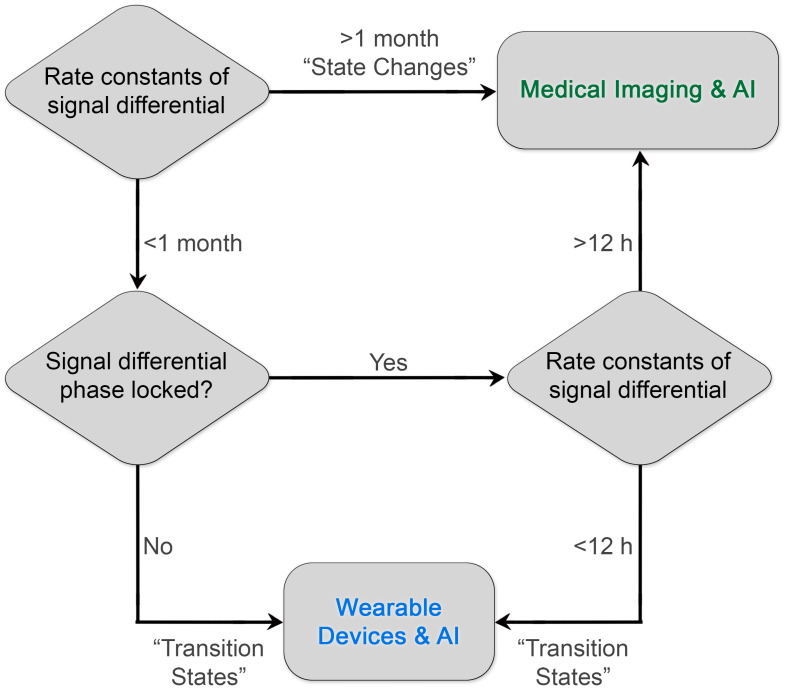
Diagram for prioritizing the development of diagnostics. By matching the rate constants of the diagnostic biological information to the frequency of reliable sampling of the clinical medical imaging process, investigators can maximize the impact on patient outcomes and establish a virtuous cycle of innovation and reward. Existing examples of clinical diagnostics already illustrate this paradigm. Diagnostic processes with rate constants of signal differentials greater than 1 month include RECIST measurements of colorectal tumors. A rare but important exceptional case of phase-locked, rapid rate constants is the detection of early brain hemorrhage with contrast-enhanced CT within 12 h and ideally ≤ 20 min upon arrival in a stroke center (AHA/ASA guidelines) [53] to enable the appropriate selection of lytic therapy. Another current example of leveraging rapid differential rate constants is the use of wearable devices that monitor insulin levels and change insulin delivery.

By prioritizing low-frequency predictive processes or “state changes”, imaging researchers may improve the “hit rate” of research programs designed to identify high-value imaging-based diagnostics. Critically, high-frequency diagnostic information need not be ignored; these processes are simply better interrogated through continuous monitoring coupled with machine learning and artificial intelligence, e.g., wearable devices (Figure 2). Furthermore, existing literature around rate constants of biochemical pathways and their qualitative predictive value can be leveraged by researchers to accelerate the development and design of novel clinical diagnostics with maximal patient impact. We believe that there are other slowly varying but predictive molecular and structural targets, such as fibrotic proteins and associated tertiary and quaternary structure, or novel methods of interrogating known targets that can improve patient outcomes through early intervention or improved selection of therapy. 

## Abbreviations

MDPI	Multidisciplinary Digital Publishing Institute
MRA	Multi-Resolution Analysis
AI	Artificial Intelligence
LD	Linear dichroism

## References

[1] Cohen R., Harel D. (2007). Explaining a complex living system: Dynamics, multi-scaling and emergence. J. R. Soc. Interface.

[2] van den Berg N.I., Machado D., Santos S., Rocha I., Chacón J., Harcombe W., Mitri S., Patil K.R. (2022). Ecological modelling approaches for predicting emergent properties in microbial communities. Nat. Ecol. Evol..

[3] Pepe M.S., Feng Z., Janes H., Bossuyt P.M., Potter J.D. (2008). Pivotal evaluation of the accuracy of a biomarker used for classification or prediction: Standards for study design. J. Natl. Cancer Inst..

[4] Gammon S.T., Engel B.J., Gores G.J., Cressman E., Piwnica-Worms D., Millward S.W. (2020). Mistiming Death: Modeling the Time-Domain Variability of Tumor Apoptosis and Implications for Molecular Imaging of Cell Death. Mol. Imaging Biol..

[5] Palard-Novello X., Blin A.L., Le Jeune F., Garin E., Salaün P.Y., Devillers A., Gambarota G., Querellou S., Bourguet P., Saint-Jalmes H. (2018). Optimization of temporal sampling for (18)F-choline uptake quantification in prostate cancer assessment. EJNMMI Res..

[6] Kaleva E., Toyras J., Jurvelin J.S., Viren T., Saarakkala S. (2009). Effects of ultrasound frequency, temporal sampling frequency, and spatial sampling step on the quantitative ultrasound parameters of articular cartilage. IEEE Trans. Ultrason. Ferroelectr. Freq. Control.

[7] Renner K.E., Peebles A.T., Socha J.J., Queen R.M. (2022). The impact of sampling frequency on ground reaction force variables. J. Biomech..

[8] Fallahtafti F., Wurdeman S.R., Yentes J.M. (2021). Sampling rate influences the regularity analysis of temporal domain measures of walking more than spatial domain measures. Gait Posture.

[9] Holdaway D., Yang Y. (2016). Study of the Effect of Temporal Sampling Frequency on DSCOVR Observations Using the GEOS-5 Nature Run Results (Part I): Earth’s Radiation Budget. Remote Sens..

[10] Babu M., Franciosa P., Ceglarek D. (2019). Spatio-Temporal Adaptive Sampling for effective coverage measurement planning during quality inspection of free form surfaces using robotic 3D optical scanner. J. Manuf. Syst..

[11] Cheng Y., Wei Y., Liao H. (2022). Optimal sampling-based sequential inspection and maintenance plans for a heterogeneous product with competing failure modes. Reliab. Eng. Syst. Saf..

[12] Park D., Don A.S., Massamiri T., Karwa A., Warner B., MacDonald J., Hemenway C., Naik A., Kuan K.-T., Dilda P.J. (2011). Noninvasive imaging of cell death using an Hsp90 ligand. J. Am. Chem. Soc..

[13] Smith B.A., Gammon S.T., Xiao S., Wang W., Chapman S., McDermott R., Suckow M.A., Johnson J.R., Piwnica-Worms D., Gokel G.W. (2011). In vivo optical imaging of acute cell death using a near-infrared fluorescent zinc-dipicolylamine probe. Mol. Pharm..

[14] Tolar M., Abushakra S., Hey J.A., Porsteinsson A., Sabbagh M. (2020). Aducanumab, gantenerumab, BAN2401, and ALZ-801-the first wave of amyloid-targeting drugs for Alzheimer’s disease with potential for near term approval. Alzheimers Res. Ther..

[15] Zhao N., Bardine C., Lourenço A.L., Wang Y.-H., Huang Y., Cleary S.J., Wilson D.M., Oh D.Y., Fong L., Looney M.R. (2021). In Vivo Measurement of Granzyme Proteolysis from Activated Immune Cells with PET. ACS Cent. Sci..

[16] Pisaneschi F., Gammon S.T., Paolillo V., Qureshy S.A., Piwnica-Worms D. (2022). Imaging of innate immunity activation in vivo with a redox-tuned PET reporter. Nat. Biotechnol..

[17] Zhang J., Ni R., Oke I., Calabrese C., Strouse J., Weinmann S., Ladouceur A. (2024). Imaging in Rheumatic Immune-related Adverse Events. Rheum. Dis. Clin. N. Am..

[18] Haubner F., Ohmann E., Pohl F., Strutz J., Gassner H.G. (2012). Wound healing after radiation therapy: Review of the literature. Radiat. Oncol..

[19] van der Vegt S.A., Wang Y.J., Polonchuk L., Wang K., Waters S.L., Baker R.E. (2022). A model-informed approach to assess the risk of immune checkpoint inhibitor-induced autoimmune myocarditis. Front. Pharmacol..

[20] Reinke J.M., Sorg H. (2012). Wound repair and regeneration. Eur. Surg. Res..

[21] Dulfer E.A., Joosten L.A.B., Netea M.G. (2024). Enduring echoes: Post-infectious long-term changes in innate immunity. Eur. J. Intern. Med..

[22] Moss B.J., Ryter S.W., Rosas I.O. (2022). Pathogenic Mechanisms Underlying Idiopathic Pulmonary Fibrosis. Annu. Rev. Pathol..

[23] Moser C.C., Farid T.A., Chobot S.E., Dutton P.L. (2006). Electron tunneling chains of mitochondria. Biochim. Biophys. Acta.

[24] Bootman M.D., Bultynck G. (2020). Fundamentals of Cellular Calcium Signaling: A Primer. Cold Spring Harb. Perspect. Biol..

[25] Fojt O., Holcik J. (1998). Applying nonlinear dynamics to ECG signal processing. IEEE Eng. Med. Biol. Mag..

[26] Daubechies I. (1988). Orthonormal bases of compactly supported wavelets. Commun. Pure Appl. Math..

[27] (2024). Sampling and Multiresolution Analysis. (Rijksuniversiteit Groningen), p Book Chapter Describing MRA and Sampling Requirements. https://pure.rug.nl/ws/portalfiles/portal/3266733/c4.pdf.

[28] Serina M.S., Mosin S.G. Digital Audio Information Compression using Wavelets. Proceedings of the International Conference Mixed Design of Integrated Circuits and Systems.

[29] Beyer T., Czernin J., Freudenberg L.S. (2011). Variations in clinical PET/CT operations: Results of an international survey of active PET/CT users. J. Nucl. Med..

[30] Clover B. (2024). Top trust warns GPs of 15-week wait for scans. HSJ.

[31] Bolin S., Nilsson E., Sjodahl R. (1983). Carcinoma of the colon and rectum—growth rate. Ann. Surg..

[32] Dervan P.B. (2016). Ahmed H. Zewail (1946–2016). Science.

[33] Wang Z., Wu Y., Li X., Bai Y., Chen H., Ding J., Shen C., Hu Z., Liang D., Liu X. (2022). Comparison between a dual-time-window protocol and other simplified protocols for dynamic total-body (18)F-FDG PET imaging. EJNMMI Phys..

[34] Besson F.L., Faure S. (2024). PET KinetiX-A Software Solution for PET Parametric Imaging at the Whole Field of View Level. J. Imaging Inform. Med..

[35] Witney T.H., Kettunen M.I., Hu D.-E., Gallagher F.A., Bohndiek S.E., Napolitano R., Brindle K.M. (2010). Detecting treatment response in a model of human breast adenocarcinoma using hyperpolarised [1-13C]pyruvate and [1,4-13C2]fumarate. Br. J. Cancer.

[36] Dutta P., Pando S.C., Mascaro M., Riquelme E., Zoltan M., Zacharias N.M., Gammon S.T., Piwnica-Worms D., Pagel M.D., Sen S. (2020). Early Detection of Pancreatic Intraepithelial Neoplasias (PanINs) in Transgenic Mouse Model by Hyperpolarized (13)C Metabolic Magnetic Resonance Spectroscopy. Int. J. Mol. Sci..

[37] Hood D.C., Kardon R.H. (2007). A framework for comparing structural and functional measures of glaucomatous damage. Prog. Retin. Eye Res..

[38] Muthu Krishnammal P., Raja S.S. Deep Learning Based Image Classification and Abnormalities Analysis of MRI Brain Images. Proceedings of the 2019 TEQIP III Sponsored International Conference on Microwave Integrated Circuits, Photonics and Wireless Networks (IMICPW).

[39] Mecheter I., Abbod M., Amira A., Zaidi H. (2022). Deep learning with multiresolution handcrafted features for brain MRI segmentation. Artif. Intell. Med..

[40] Hernstrom V., Josefsson V., Sartor H., Schmidt D., Larsson A.-M., Hofvind S., Andersson I., Rosso A., Hagberg O., Lång K. (2025). Screening performance and characteristics of breast cancer detected in the Mammography Screening with Artificial Intelligence trial (MASAI): A randomised, controlled, parallel-group, non-inferiority, single-blinded, screening accuracy study. Lancet Digit. Health.

[41] Didyuk O., Econom N., Guardia A., Livingston K., Klueh U. (2021). Continuous Glucose Monitoring Devices: Past, Present, and Future Focus on the History and Evolution of Technological Innovation. J. Diabetes Sci. Technol..

[42] (2022). Multiresolution and Deep Neural Networks. Wavelets in Soft Computing.

[43] Shin N., Park J. (2018). The Effect of Intentional Nursing Rounds Based on the Care Model on Patients’ Perceived Nursing Quality and their Satisfaction with Nursing Services. Asian Nurs. Res. (Korean Soc. Nurs. Sci.).

[44] Levin M.A., Kia A., Timsina P., Cheng F.-Y., Nguyen K.-A., Kohli-Seth R., Lin H.-M.S., Ouyang Y., Freeman R.R., Reich D.L. (2024). Real-Time Machine Learning Alerts to Prevent Escalation of Care: A Nonrandomized Clustered Pragmatic Clinical Trial. Crit. Care Med..

[45] Johnson C., Mathew S.J., Oh H., Rufino K.A., Najafi B., Colombo A.E., Patriquin M.A. (2021). Wearable technology: A promising opportunity to improve inpatient psychiatry safety and outcomes. J. Psychiatr. Res..

[46] (2025). OWASP Top 10. https://owasp.org/www-project-top-ten/.

[47] (2025). NVD. https://nvd.nist.gov/.

[48] Jaime F.J., Munoz A., Rodriguez-Gomez F., Jerez-Calero A. (2023). Strengthening Privacy and Data Security in Biomedical Microelectromechanical Systems by IoT Communication Security and Protection in Smart Healthcare. Sensors.

[49] Mejia-Granda C.M., Fernandez-Aleman J.L., Carrillo-de-Gea J.M., Garcia-Berna J.A. (2024). Security vulnerabilities in healthcare: An analysis of medical devices and software. Med. Biol. Eng. Comput..

[50] Kjelle E., Brandsæter I.Ø., Andersen E.R., Hofmann B.M. (2024). Cost of Low-Value Imaging Worldwide: A Systematic Review. Appl. Health Econ. Health Policy.

[51] Miao Y., Jin J., Daly I., Zuo C., Wang X., Cichocki A., Jung T.-P. (2021). Learning Common Time-Frequency-Spatial Patterns for Motor Imagery Classification. IEEE Trans. Neural Syst. Rehabil. Eng..

[52] Hong N., Kim B., Lee J., Choe H.K., Jin K.H., Kang H. (2024). Machine learning-based high-frequency neuronal spike reconstruction from low-frequency and low-sampling-rate recordings. Nat. Commun..

[53] Powers W.J., Rabinstein A.A., Ackerson T., Adeoye O.M., Bambakidis N.C., Becker K., Biller J., Brown M., Demaerschalk B.M., Hoh B. (2019). Guidelines for the Early Management of Patients With Acute Ischemic Stroke: 2019 Update to the 2018 Guidelines for the Early Management of Acute Ischemic Stroke: A Guideline for Healthcare Professionals From the American Heart Association/American Stroke Association. Stroke.

